# Effects of Managed and Unmanaged Floral Margins on Pollination Services and Production in Melon Crops

**DOI:** 10.3390/insects14030296

**Published:** 2023-03-20

**Authors:** María Pérez-Marcos, Francisco Javier Ortiz-Sánchez, Elena López-Gallego, Helena Ibáñez, Aline Carrasco, Juan Antonio Sanchez

**Affiliations:** 1 Biological Pest Control & Ecosystem Services Laboratory, Institute of Agricultural and Environmental Research and Development (IMIDA), C/Mayor s/n, 30150 Murcia, Spain; 2 Research Group “R&D Transfer in the Area of Natural Resources”, University of Almería, Ctra. de Sacramento s/n, La Cañada de San Urbano, 04120 Almería, Spain

**Keywords:** agroecosystems, bees, crop pollination, ecosystem services, floral margins, herbaceous margins, melon crops, pollination service, shrubby margins

## Abstract

**Simple Summary:**

Improving floral margins around crops can help to maintain and conserve populations of wild pollinators, mainly insects, in agroecosystems, and can improve the ecosystem services they provide, such as pollination of many crops and wild plants. Some crops, such as melon, depend on insects for reproduction, which is why they are at risk, as pollination services are declining. Our study shows that the implementation of margins around melon crops showed a positive effect on the abundance and richness of pollinating insects in general, which was stronger in the second year after establishment. Specifically, managed shrubby margins favored the populations of Syrphidae, Andrenidae, Apidae (excl. *Apis mellifera*), and other pollinator groups, and bee richness, suggesting that this type of margin increases pollinators’ activity to a greater extent than the other two types of margins (managed and unmanaged herbaceous margins). However, the type of margin did not show any added advantages for melon yield.

**Abstract:**

Melon is among the most consumed fruits in the world, being a crop that depends almost entirely on insects for its reproduction, which is why it is especially sensitive to declining pollination services. Restoration and maintenance of hedgerows and agricultural borders around crops are generally carried out by sowing flowering herbaceous plants or establishing shrubby species; however, a cost-effective and lower-maintenance alternative for farmers could be as simple as allowing vegetation to regenerate naturally without any management actions. This work aimed to test the effects of three different types of margins (managed herbaceous, managed shrubby, and unmanaged herbaceous) on the overall abundance and richness of wild pollinators in melon crops. The work was performed in three localities in southern Spain over two years. Pollinators were monitored visually using 1 × 1 m sampling squares and pan traps within melon fields. Moreover, crop yield was estimated by measuring fruit weight and the number of seeds. In general, higher abundances of pollinators were observed in melon fields during the second year. In addition, the abundances of Syrphidae, Andrenidae, Apidae (excl. *Apis mellifera*), and pollinators other than bees, belonging to the orders Diptera, Coleoptera, Hymenoptera, and Lepidoptera, showed higher values in melon fields with shrubby margins than in fields with herbaceous margins (managed or unmanaged). However, no effect of floral margins on the yield of melon crops was found.

## 1. Introduction

Insects are key components of biodiversity and provide important ecosystem services, as in the case of those that act as pollinators of many crops and wild plants [1,2,3,4]. It is estimated that pollinators affect 35% of the world’s agricultural land and support the production of 87% of the world’s major food crops [5], affecting both their quality and quantity. More specifically, some important world crops such as melon, zucchini, and apple are almost entirely dependent on pollinating insects [5]. However, we are witnessing a global decline in the abundance and diversity of pollinators [1,3,6,7]. In Europe, with a severe lack of data, it has been estimated that, overall, 9% of bee and butterfly species are threatened, and that regarding the best-known bee species (which represent only 47% of the total), their populations are declining by 37% [3].

Land-use changes, soil degradation, landscape homogenization, habitat degradation and fragmentation, intensification of agriculture and its management, and the extensive use of pesticides are some of the main pressures that pollinators suffer at different spatial and temporal scales [3,8,9,10,11,12]. Thus, the high pressure suffered by pollinators in these agricultural systems threatens the pollination service. For this reason, most entomophilous crops suffer production limitations, not reaching the maximum production because they do not receive the maximum necessary contributions of pollen from insects [13]. The quantity and quality of crop pollination depend on the abundance and richness of specific pollinators, which are related to the environmental complexity at the landscape scale (quantity and connectivity of natural or semi-natural habitats) and the management of vegetation at the plot scale [14,15].

Enhancing floral resources is a frequently adopted strategy to manage the loss of biodiversity in agricultural landscapes [16,17,18,19]. Floral margins in cultivated fields can provide both continuous floral resources beyond the crop bloom interval and suitable nesting sites for different species. In addition, they are important landscape elements that connect habitat fragments and alleviate the negative effects of habitat loss and isolation on biodiversity [19,20,21,22,23,24]. Several authors have investigated the use of these flowering margins to ameliorate the losses of functional diversity, showing an increase in the abundance and diversity of wild and managed pollinating insects [16,19,25,26,27,28,29,30,31,32,33,34]. In this sense, studies and experiences normally focus on works of establishment or restoration of field margins carried out by sowing herbaceous flowering plants [18,32], or establishing shrubby species, giving minor consideration to the role of unmanaged margins [19,24,34]. These different types of managed margins—herbaceous or shrubby—have requirements (irrigation, mowing and resowing, pruning, etc.) to avoid a decrease in the density and diversity of flowers in successive seasons [18,27,34,35]. However, a lower-maintenance, cost-effective alternative could be to simply allow the vegetation to regenerate naturally without any maintenance [19].

It is also important to point out that although the floral margins support a greater abundance and diversity of pollinators than just crops [35,36], only some works have examined whether improving floral resources generates an effect of concentration or export of pollinating insects to adjacent crops [37,38,39,40,41,42], which is also a great concern for farmers [36,43]. Although some studies suggest that margins improve rather than reduce pollination services in nearby crops, the current evidence is not conclusive, and the results vary by crop type [23,40,41,44,45,46].

The main goal of this study was to assess the effect of three different types of floral margins on the abundance, diversity, and community composition of melon-pollinating insects in an area of intensive agriculture. The melon crop, *Cucumis melo* L., 1753 (Cucurbitaceae), requires insect pollination [47], and on a regional scale, in order to optimize this process, crops are sometimes reinforced with the placement of *Apis mellifera* Linnaeus, 1758 hives in their surroundings to improve the quality and quantity of fruits [48]. However, it is important to bear in mind that wild bees and other wild pollinators can provide equal and/or complementary services compared to those of managed bees [39,49]. Therefore, we established two melon field margins with managed flowering plant species that were previously tested to improve bee communities in areas of intensive agriculture [34] and compared them with melon fields where vegetation was allowed to regenerate naturally without maintenance on their edges. We then determined the abundance and diversity of pollinating insects on the melon fields and the melon yield during a period of two years.

Specifically, we asked the following questions: Do wild pollinator abundance, bee abundance, and bee richness in melon crops differ depending on the associated floral margin type? Does crop yield differ depending on the type of margin associated with the crop?

## 2. Materials and Methods

### 2.1. Study Area

The assay was carried out in 2014 and 2015 on three experimental farms in the Region of Murcia (SE of Spain) (Table 1). The localities were separated from one another by at least 7 km. All of them were surrounded by diverse horticultural crops and scattered spaces of ruderal vegetation [50]. The climate of the area is characterized by high temperatures, with an important daily variability and scarce rainfall. The mean temperature in the ten-year period 2005–2015 was 18 °C, with an average maximum of 30 °C and an average minimum of 5 °C. In this period, the annual precipitation ranged between 198 and 437 mm (Sistema de Información Agrario de Murcia).

### 2.2. Design and Setting of the Experiment

In each locality, three melon plots of approximately 300 m^2^ were delimited. Each plot, which was planted with green-type melon, had 5 lines of 30–35 plants, with 2 m separation between lines and 1 m between plants within lines. Melon fields were sprayed when it was utterly necessary and only to treat fungal diseases, and no insecticides were used. The melon plants were transplanted in both years at the beginning of April, and fruits were harvested from mid-June to the beginning of July.

For each melon field, a margin was established perpendicularly at a distance of one meter from the edge of the crop. Three types of margins were placed per locality: (1) margin sown with a mixture of herbaceous plants (MH) that belonged to different families (Table S1, Supplementary Material); (2) margin revegetated with shrubby plants (MS), integrated mainly with Lamiaceae species (Table S1, Supplementary Material); and (3) unmanaged herbaceous margin (UH), with naturally emerged herbaceous plants (Table S1, Supplementary Material). The margin strips had a surface of 100 m^2^ (20 m × 5 m). The herbaceous seed mixture was sown manually in the autumns of 2013 and 2014. Shrubs were transplanted in the autumn of 2013. Managed margins were irrigated once every one or two weeks and the shrubby margins were weeded periodically. The unmanaged margins remained without any intervention throughout the study period.

### 2.3. Sampling of Bees

The abundance of bees and other pollinators visiting the melon crops was estimated by visual sampling and pan trap sampling. Pollinators were grouped into eight categories: (1) *Apis mellifera*, (2) Apidae, (3) Andrenidae, (4) Colletidae, (5) Halictidae, (6) Megachilidae, (7) Syrphidae, and (8) other pollinators (Coleoptera, other Diptera, other Hymenoptera, and Lepidoptera). Apidae includes all the genera of the family Apidae except *Apis mellifera*.

Visual samplings were carried out by counting the number of bees within a 1 m × 1 m square during a 5 min period. The squares were placed six times at increasing distances (5, 10, 15, 20, 25, and 35 m) from the floral margins. Samplings were performed from 09:00 to 16:00 in sunny conditions with a temperature above 20 °C and low wind speed. Given the short duration of the crop, only two visual samplings were carried out each year, from weeks 22 to 23 (end of May/beginning of June) in 2014 and weeks 21 to 22 (mid-to-late May) in 2015. Therefore, the community of pollinators of the melon crop associated with different margins (3) in each locality (3) was visually sampled on 4 occasions throughout the two years of study, leading to a total of 216 sampled quadrats. Bees were identified at the genus level; however, a few were only identified at the family level due to the difficulties that can sometimes be found in visual samplings.

The yellow pan traps were 28 cm in diameter and 14 cm high and were filled with water, formaldehyde (0.1%), and a drop of detergent. The yellow color was used because it is known that it collects a greater richness of bee species [39,51]. Six pan traps were placed per crop at six different distances from the floral margin (5, 10, 15, 20, 25, and 35 m). The traps were placed from weeks 20 to 28 (mid-May to the beginning of July) in both years, being emptied every week. The specimens collected were preserved in 70% ethanol until they were dried and mounted for their identification. This procedure was repeated for each of the three melon crops in each locality. Bees were identified at the genus level. The voucher specimens were submitted to the Institute of Agricultural and Environmental Research and Development (IMIDA, Murcia, Spain).

### 2.4. Harvest and Yield

To determine the average production and the quality of the fruits, all melons from five plants were collected for each of the six distances previously established. The melons were transferred to the laboratory where they were weighed and measured (length and width). The largest melon on each plant was selected. After being measured in the laboratory, the seeds were extracted and dried at room temperature for approximately 5 days, when they were counted and weighted.

### 2.5. Analysis of Data

#### 2.5.1. Relative Pollinators Abundance and Richness in Melon Fields Depending on the Type of Margin

Generalized mixed effect models (GLMMs) were used to test the effect of the margin type, the year, and the distance from the margin on the abundance of *Apis mellifera* and wild bees, other pollinators, and Syrphidae recorded in the melon crops in visual samplings. For the analysis of bee abundance, wild bee families were grouped due to their low values of abundance. The margin type, year, and distance were introduced in the models as fixed factors, and the locality and sampling date were introduced as random factors. Although the effect of the margin on the pollinator community should be more or less the same over the years, the interaction “margin type × year” was included in the model since the margin structure may change because of the recent establishment of the margins. In addition, as different distance slopes between each margin type may be expected, an interaction “margin type × distance” was added to the model.

The same above-explained analytical approach was followed to test the effect of the margin type, year, and distance from the margin, as fixed factors, on the number of captures of the different groups of bees (i.e., *Apis mellifera*, Apidae, Andrenidae, Colletidae, Halictidae, and Megachilidae), other pollinators, and Syrphidae found in the pan trap sampling. In both analyses, the GLMMs were set to the negative binomial [52].

In addition, to determine the effects of the different types of margins, years, and distances from the margin, as fixed factors, on the richness of bee genera collected in the pan traps, another GLMMs was run. The locality and sampling date were introduced in the model as random factors. In this case, the GLMMs was set to a Gaussian distribution with the link “‘log”’.

In all analyses, the GLMMs were run using the function “glmmPQL” (“MASS package”) [53]. The goodness of fit for the distribution was tested using the function “fitdist” in the R “fitdistrplus” package [54]. The χ^2^ and *p*-values for the fixed factors were obtained through the Wald test using the “Anova” function in the R “car” package [52]. The contrast for the abundance of the defined groups of pollinating insects and the richness of bee genera among the melon fields with the different margin types was tested using the Tukey test with the function “glht” in the “multcomp” package [55].

#### 2.5.2. Analyses of Crop Production

The melon productivity (weight and number of seeds) of the crops was analyzed through one-way analysis of variance (ANOVA) to test the effect of the different margin types. The means of each treatment were separated with the LSD test (α = 0.05) [56].

## 3. Results

### 3.1. Some Considerations on the Floral Resources Provided by the Margins

The three margins differed in flower coverage, and the richness and diversity of flowering plants that they presented. The herbaceous margins presented higher abundance values, followed by the shrubby margins and, finally, with significantly lower values, the unmanaged herbaceous margins. Significant differences were observed between years in flower coverage in the shrubby margins, with a significantly higher flower coverage in the second year compared to the first (Pérez-Marcos et al., unpublished).

### 3.2. Abundance of Pollinators in Melon Crops Depending on the Floral Margin Type in Visual Samplings

During the visual samplings, 159 pollinating insects were observed. Of these, 86.2% were bees, followed by other pollinator groups (10.7%), and Syrphidae (3.1%) (Table 2). Among the different groups of bees, *Apis mellifera* accounted for 50.3% of the total pollinators observed and wild bees accounted for 35.8%. Among the wild bees, Halictidae (15.1% of the total pollinators), Megachilidae (10.7%), and Apidae (excl. *Apis mellifera*) (8.18%) were the main families visiting melon flowers (Table 2). Very few individuals of the families Andrenidae (1.3%) and Colletidae (0.6%) were observed.

*Apis mellifera* abundance was not found to be significantly influenced by the type of margin (χ^2^ = 0.061, df = 2, *p* = 0.970), year (χ^2^ = 1.429, df = 1, *p* = 0.232), or distance (χ^2^ = 0.0, df = 1, *p* = 0.998). This species had similar abundances in all melon fields, independent of the type of margin, with values varying between 1.4 ± 0.4 individuals/m^2^ ± SE in the crops with managed herbaceous margins and 1.0 ± 0.4 individuals/m^2^ ± SE in the crops with unmanaged herbaceous margins. In the second year, lower abundances of this species were registered in all crops, with the same pattern as the first year (managed herbaceous: 0.7 ± 0.5 individuals/m^2^ ± SE; unmanaged herbaceous: 0.5 ± 0.2 individuals/m^2^ ± SE).

Wild bee abundances, considering all the families grouped due to their low abundances, were not significantly influenced by the type of margin (χ^2^ = 5.943, df = 2, *p* = 0.051), the year (χ^2^ = 3.111, df = 1, *p* = 0.078), or the distance (χ^2^ = 5.943, df = 2, *p* = 0.051). The abundance of wild bees was significantly higher in the melon fields with managed herbaceous margins than in those with shrubby margins (*p* = 0.032), but no significant differences between shrubby and unmanaged herbaceous margins or between managed and unmanaged herbaceous margins were found (Tukey contrast, *p* = 0.701 and *p* = 0.179).

The abundance of other pollinators was not influenced by the type of margin (χ^2^ = 5.562, df = 2, *p* = 0.062), or the year (χ^2^ = 0.02, df = 1, *p* = 0.895) or the distance (χ^2^ = 0.053, df = 1, *p* = 0.818). In both years, its maximum abundance was registered in crops with managed herbaceous margins (0.4 ± 0.2 individuals/m^2^ and 0.3 ± 0.1 individuals/m^2^, respectively) but no significant differences were observed when comparing melon fields with the other two margins (Tukey contrast, MH vs. MS: *p* = 0.2124 and MH vs. UH: *p* = 0.0624).

The abundance of Syrphids was low and not significantly influenced by the type of margin (χ^2^ = 0.481, df = 2, *p* = 0.786), the year (χ^2^ = 0.918, df = 1, *p* = 0.338), or the distance (χ^2^ = 1.899, df = 1, *p* = 0.168).

### 3.3. Abundance of Pollinating Insects in Melon Fields Depending on the Floral Margin Type in Pan Traps

During the two years of the study, 2681 insects were captured in the pan traps. Among them, 68.1% were bees, while 31% were other pollinators, and 8.7% were Syrphidae. The remaining 0.7% were occasional insects (Table 3).

The GLMM analysis showed that, in this case, the abundance of *Apis mellifera* and Apidae was significantly influenced by the type of margin, but this was not the case for the families Halictidae, Andrenidae, Colletidae and Megachilidae. Halictidae and Colletidae abundances were influenced by the year, registering higher abundances in the second year compared with the first in both cases. *Apis mellifera* and Andrenidae abundances were influenced by the interaction margin type × year, and Andrenidae and Apidae were influenced by the distance from the margin with a negative relationship (Table S2, Supplementary Material).

**Figure 1:**
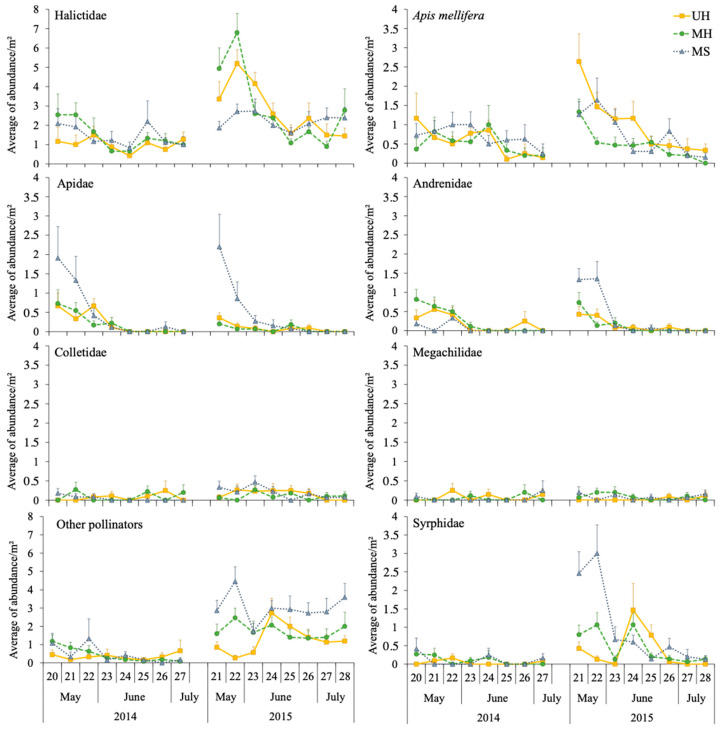
Average (±SE) abundances of the different bee families, other pollinators, and Syrphidae on each sampling date (week number) in the pan trap samplings of crops with shrubby (MS), managed, and unmanaged herbaceous margins (MH and UH, respectively).

In the first year, Halictidae, Apidae, and Colletidae had similar abundances in all melon fields, independent of the type of margin. However, Andrenidae abundances were significantly higher in melon fields with managed and unmanaged herbaceous margins than in those with shrubby margins (Tukey contrast, *p* < 0.01), but the opposite occurred for Apidae, with significantly higher abundances in fields with shrubby margins than in those with herbaceous margins (Tukey contrast; *p* < 0.001) (Figure 1). In the second year, Halictidae and Colletidae dynamics were similar in all three melon fields but with higher abundances compared with the first year. *Apis mellifera* abundance was significantly higher in melon fields with unmanaged herbaceous margins than in those with managed herbaceous margins (Tukey contrast; *p* < 0.001). Furthermore, Andrenidae and Apidae had significantly higher abundances in melon fields with shrubby margins than in fields with herbaceous margins (Tukey contrast, *p* < 0.01). Megachilidae was registered with very low abundances in all three fields in both years and although the interaction margin type × year was significant, there were no significant differences found in the contrast interaction (Tukey contrast; *p* > 0.05) (Figure 1).

The abundance of other pollinating insects was significantly influenced by the type of margin (χ^2^ = 29, df = 2, *p* < 0.001), the year (χ^2^ = 64.7, df = 1, *p* < 0.001), and the interaction between them (χ^2^ = 6.7, df = 2, *p* = 0.034). The abundances recorded in the melon fields were significantly higher during the second year (Tukey contrast, *p* < 0.001). Moreover, in 2015, other pollinator abundances in the fields with shrubby margins were significantly higher than in crops with herbaceous margins (managed and unmanaged) (Tukey contrast, *p* < 0.001) (Figure 1).

Syrphidae abundance in melon fields was influenced by the type of margin (χ^2^ = 21.3, df = 2, *p* < 0.001) and the year (χ^2^ = 23.5, df = 1, *p* < 0.001), but no differences were found in their interaction or in the distance from the margin (χ^2^ = 1.4, df = 2, *p* = 0.486 and χ^2^ = 0.3, df = 1, *p* = 0.568, respectively). During the second year, abundances were significantly higher compared with the first year (Tukey contrast, *p* < 0.01), and fields with shrubby margins had significantly higher abundances than fields with unmanaged herbaceous margins (Tukey contrast, *p* < 0.001), but no differences were observed between shrubby and managed herbaceous margins or between both herbaceous margins (Tukey contrast, *p* > 0.05) (Figure 1).

### 3.4. Bee Genera Richness in Melon Crops Depending on the Margin Type

During the two years of the study, 137 bees were observed through visual sampling, identifying 5 families and 14 genera. The main genera included *Apis* (58.4% of the bees observed), *Lasioglossum* (15.3%), *Megachile* (6.6%), *Ceratina* (6.6%), and *Anthidium* (2.9%), accounting for around 90% of the bee visits recorded in the visual square samplings, with the remaining nine bee genera accounting for 10.2% of the bee visits (Table 2). Moreover, 1827 bees were recorded in the pan trap samplings, registering a total of 25 genera. *Lasioglossum* (51.5% of the bees recorded), together with the genera *Apis* (19.1%), *Eucera* (7.6%), *Andrena* (5.4%), *Halictus* (4.6%), *Nomioides* (4.5%), and *Hylaeus* (3.0%), represented about 95.6% of the specimens captured in the pan traps. The remaining 17 genera represented 4.4% of the total insects recorded in the pan traps, with all of them being below 1% of the specimens registered (Table 3).

**Figure 2:**
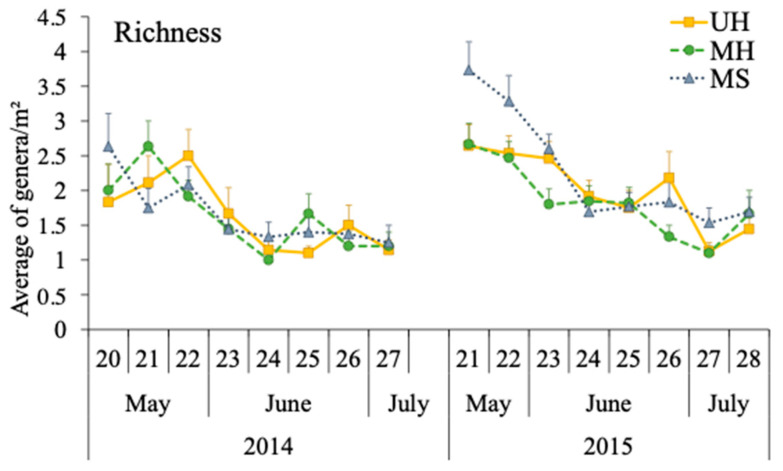
Average (±SE) richness of the different bee genera per melon field on each sampling date (week number) in the pan trap samplings in melon fields with shrubby (MS), managed, and unmanaged herbaceous margins (MH and UH, respectively).

The richness recorded in the pan traps was significantly influenced by the type of margin (χ^2^ = 10.6, df = 2, *p* = 0.005), the year (χ^2^ = 4.1, df = 2, *p* = 0.044), and the treatment × year interaction (χ^2^ = 7.7, df = 2, *p* = 0.021). In the first year, no significant differences were observed between the three melon fields (Tukey contrast, *p* = 0.99), but the number of bee genera was significantly higher in the fields with shrubby margins than in both fields with herbaceous margins in the second year (Tukey contrast, MS vs. MH: *p* < 0.01; MS vs. UH: *p* = 0.04). No significant differences were observed between both melon fields with herbaceous margins (Tukey contrast, *p* = 0.9) (Figure 2). Bee genus richness followed similar patterns in all three melon fields during the first year, peaking consecutively throughout May (fields with managed shrubby margins: 2.6 ± 1.6 genera/sampling ± SE in the second week; managed herbaceous margins: 2.6 ± 1.2 genera/sampling ± SE in the third week; unmanaged herbaceous margins: 2.5 ± 1.3 genera/sampling ± SE in the fourth week). During the second year, the dynamics of both melon fields with herbaceous margins were the same, recording the same number of genera as in the first year (both: 2.6 ± 1.2 genera/sampling ± SE); however, melon fields with shrubby margins registered a peak in mid-May with a higher number of genera than in the first year (3.7 ± 1.6 genera/sampling ± SE) (Figure 2).

### 3.5. Effect of Floral Margin Type on Crop Production

**Figure 3:**
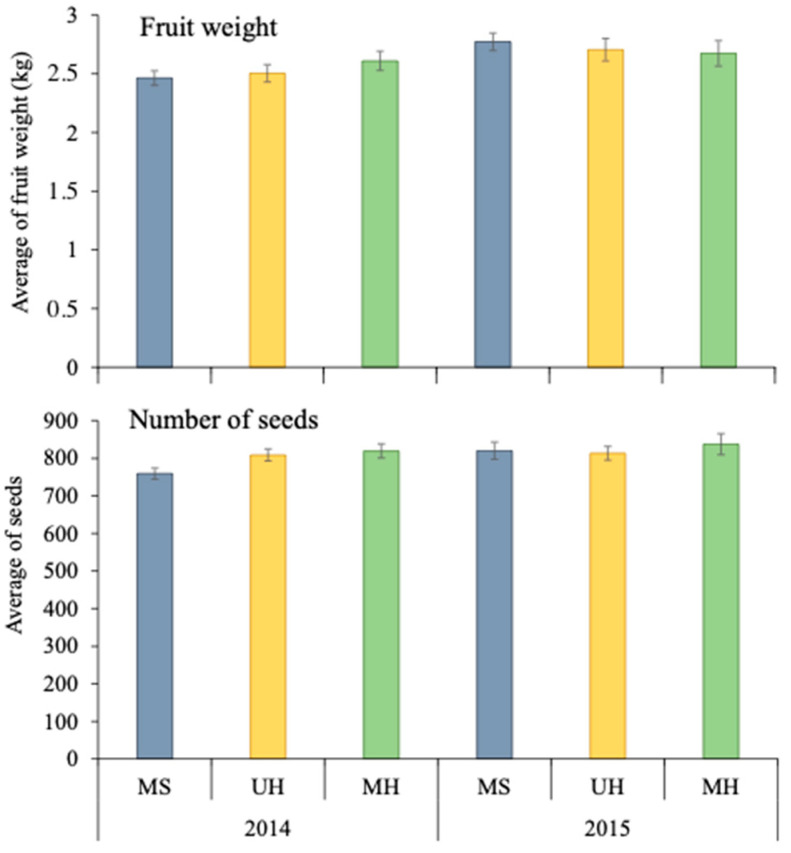
Average (±SE) fruit weight and number of viable seeds in each year in melon crops with shrubby (MS), managed, and unmanaged herbaceous margins (MH and UH, respectively).

Fruit weight was not influenced by the margin type (F = 0.37, df = 2, 353, *p* = 0.694) or by the interaction treatment × year (F = 1.14, df = 2, 353, *p* = 0.321), but it was influenced by the year (F = 8.52, df = 1, 353, *p* = 0.004), with a significantly higher weight being observed in melon fields with managed shrubby margins during the second year compared with those during the first year. A slight increase was observed in the fields with both managed and unmanaged herbaceous margins during the second year, but it was not significant (Figure 3). In the first year, the average melon weight was 2.46 ± 0.06 kg in melon fields with shrubby margins and 2.61 ± 0.08 kg in melon fields with managed herbaceous margins. In the second year, the fields with shrubby margins registered the highest average weight (2.77 ± 0.07 kg ± SE), and melon fields with managed herbaceous margins registered the lowest (2.67 ± 0.11 kg ± SE) (Figure 3).

Moreover, the number of seeds was slightly influenced by the margin type (F = 3.3, df = 2, 353, *p* = 0.039), but not by the year (F = 3.3, df = 1, 353, *p* = 0.067). The number of seeds was similar in melon fields with both herbaceous margins in the first year (MH: 819.4 ± 19.1 seeds; UH: 808.6 ± 16.1 seeds), and a significantly lower number of seeds were found in fields with managed shrubby margins (758.3 ± 14.6 seeds). In the second year, the three melon fields had a similar number of seeds, between 813.1 ± 18.3 and 836.9 ± 28.0 seeds ± SE (Figure 3).

## 4. Discussion

### 4.1. Effect of Floral Margins Types Associated with Melon Crops on the Abundance and Richness of Insects

The overall objective of this work was to evaluate whether different types of floral margins could affect the abundance, diversity, and community composition of pollinating insects of melon crops. Here, we show that the type of margin had a significant effect on the abundance of some pollinator groups and on the richness of bees in the melon fields. Specifically, the abundances of Syrphidae, Andrenidae, and Apidae (excl. *Apis mellifera*) and other pollinator groups were higher in melon fields with managed shrubby margins compared to fields with herbaceous margins (managed or unmanaged). However, there were differences between the two years, with the effect not being significant in the first year. This is not surprising considering the phenology of shrubby margins ([18]; Perez-Marcos et al.) as they need additional time to complete their establishment and provide stronger flowering. In terms of the groups that showed differences, on the one hand, the higher abundance of Andrenidae in melon fields with shrubby margins could be due to their foraging habits. They are short-tongued bees and prefer shallow flowers [26,34,57], so they may not be attracted to the shrubby margins, consequently visiting the melon flowers in greater abundance. On the other hand, very little is known about the foraging behavior of the Apidae family, syrphids, and other wild pollinators [19,33,34,58]; therefore, new studies on their habits and preferences will be even more enlightening. This greater abundance mentioned in the melon fields with shrubby margins might suggest that this type of margin could favor an increase in the activity of pollinators in the crop, increasing the abundance of some groups of pollinators to a greater extent than the other two margins. Conversely, herbaceous flower margins might not act as facilitators, exerting a possible concentration effect contrary to the desired one, since it seems that they could be much more attractive to bees than nearby melon flowers [41], either due to the similarity of their floral structure, being mainly shallow flowers, or due to the greater floral richness and floral coverage compared to the shrubby margins (Pérez-Marcos et al., unpublished).

Regarding the different bee families recorded in the melon fields, the family Halictidae was the most abundant, especially the genus *Lasioglossum*. This coincides with other works that pointed out this genus as a key pollinator of melon crops [39,41,59]. This fact is remarkable, as the pollination deficit is usually solved by farmers through *Apis mellifera* hives. Although *Apis* was frequently observed and therefore expected to play a role in melon pollination, some authors found that *Lasioglossum* species required a significantly lower mean number of visits to pollinate than honeybees in watermelon crops [60], having significantly higher pollen deposition on stigmas than honeybees [61]. Furthermore, in this regard, some studies indicate that when the pollination activity is shared between two (or more) taxa of pollinators, a higher yield is obtained compared to the same overall level of activity of a single pollinating taxon [62,63], highlighting the advantages of maintaining *Lasioglossum* species in melon-growing areas. This genus could be expected to be more abundant in herbaceous margins due to its short tongue [34], being especially related to the Asteraceae family [64] and plants such as *Coriandrum sativum* [26,41]. All these types of plant species were present in both herbaceous margins adjacent to our melon crops. However, if herbaceous margins might not act as facilitators, a lower halictid abundance would be seen in the crop. However, it is worth noting that no differences were observed between the visits of *Lasioglossum* to melon fields with the three types of floral margins, with even a slightly higher abundance found in fields with managed herbaceous margins compared to the other two margins, but no significant differences. However, what was observed was an increase in the abundances of this family between the first and second years. This is in line with the results of Kleijn et al. [65], who suggest that dominant crop pollinators persist under agricultural expansion and many of them are easily enhanced by simple conservation measures.

In addition, a significant effect of the margin type on bee richness was observed. During the first year, fewer genera were associated with melon fields and no differences were observed between fields; meanwhile, during the second year, there was a considerable increase in the number of genera, with melon fields with shrubby margins showing the greatest increase. The greater richness in the fields with shrubby margins indicates that these margins favor greater pollination activity in melon crops, improving both the abundance and richness of pollinators. In particular, it was found that their effects were more evident during the second year, which matches the phenology of this type of margin, as it needs additional time to establish, creating a stronger flowering during the second year ([18] Pérez-Marcos et al., unpublished).

### 4.2. Effect of Floral Margin Type on Crop Production

Several studies have shown that the floral margin characteristics vary with the management and vegetation type [18,32], which could influence pollinators and thus crop production [42]. Our second working hypothesis was that melon yield would differ according to the type of margin. In previous studies, the response of enhancing flower strips was not conclusive. For instance, Blaauw and Isaacs [44] and Balzan et al. [45] found an increased yield in tomato and blueberry crops, but other authors have reported a lack of effect in crops such as apples, cucumbers, and melons [41,42,46,66,67]. In our case, there was no clear effect on crop yield, with a slight increase in fruit weight between years, but this was not reflected in the number of seeds, nor was it influenced by the type of margin. While this does not support our hypothesis, it does show that the margins do not reduce production. This is important because, as some authors suggest, some farmers fear that floral margins will drive pollinators away from crop fields, with a consequent decrease in pollination [68]. As mentioned, pollinators may be affected by a concentration effect on herbaceous margins, but the fact that this does not have a significant impact on crop production leads us to think that the necessary pollinator pool size for melon is sufficiently covered in our study cases. On the other hand, Kleijn et al. [65] suggest that land-use changes due to agricultural intensification affect crop production less than entomological diversity in general, as most bee species that currently provide the majority of pollination services to crops could persist, while the remaining species decline drastically in abundance. This could suggest that the dominant bee genera that visit the crops (i.e., *Lasioglossum* and *Apis*) are able to provide the pollination services required. However, we do not know whether there is room for improvement, since, as mentioned above, some authors observed a positive effect on crops only several years after establishing floral margins [44,69] and, in addition, other factors not considered in our work could affect crop production [42,67,70]; thus, longer-term studies will help us to better understand the spatio-temporal scales of the effect of floral margins on melon crop yield and on the dynamics of the associated insect populations.

## 5. Conclusions

The planting of margins around melon crops showed a positive effect on the abundance and richness of pollinating insects in general, which materialized more clearly in the second year of their establishment but did not show any added advantages for melon yield. In our case, managed shrubby margins favored the population of Syrphidae, Andrenidae, Apidae, and other pollinators groups, and bee richness, suggesting that this type of margin increases pollinator activity or attracts them to a greater extent than the other two margins. This must be put in the context of the concept of pollinator species and abundance pool size, which ultimately depends on the geographical area, environmental conditions, the type of crop, the frequent or occasional nature of pollinators, or the complexity of the landscape [70]. If this pool does not reach its maximum carrying capacity for a given crop, the effect of the export or concentration of pollinators exerted by the margins will have a clearer measurable effect on pollination and crop production.

However, it should also be considered that the effect of floral margins could favor threatened species [34] that currently contribute little to pollination ecosystem services in melon or other crops, but that should always be present in conservation strategies [65]. Moreover, it should be considered that these floral margins may require time to show their effects [68,69], and the increase in pollinators’ abundance and richness could be expected to continue even after the third year or later [42,69], suggesting that long-term studies will serve to understand this issue better.

On the other hand, it should be noted that the different sampling methodologies used in this study noted the greater capacity of traps as it captures a greater number of pollinator specimens belonging to a greater number of genera and, therefore, greater biodiversity. Moreover, Gezon et al. [71] have shown the lack of environmental impact of using pan trap under certain conditions suggesting that they are a suitable method for inventorying bee diversity and observing long-term trends in bee populations. However, this sampling method does not generate data on floral hosts and is therefore useless for studying pollination networks [72]. The visual sampling technique records lower abundances; therefore, due to the particularities of this sampling method, and as suggested by Nielsen et al. [72], in most cases, it would be necessary to increase the number of observations or the time spent on each observation to improve pollinator estimates. We also recommend advancing in the sampling and statistical design and also the training of taxonomists in the different groups of pollinators to optimize visual sampling techniques in order to detect as many species as possible and to have more conclusive results regarding the insect–pollinator–plant relationship.

## Figures and Tables

**Table 1:** Location and coordinates of the three study sites.

Locality	Nearby Town	Province	Latitude	Longitude
CIFEA	Torre Pacheco	Murcia	37°44′25.1″ N	0°58′00.1″ W
IMIDA	La Alberca	Murcia	37°56′26.2″ N	1°08′00.1″ W
Torreblanca	Dolores de Pacheco	Murcia	37°46′25.6″ N	0°53′59.7″ W

**Table 2:** List of taxa observed in visual samplings of melon crops with unmanaged herbaceous (UH), managed herbaceous (MH), and managed shrubby (MS) margins. %: percentage of observed specimens concerning the total of pollinator insects registered. Un. unidentified.

Order	Family	Genera	Treatment			Total	%
			MS	UH	MH		
Coleoptera			1	0	0	1	0.63
Diptera	Syrphidae		2	1	2	5	3.14
Hymenoptera (Apoidea)	Andrenidae	*Andrena* spp.	0	0	2	2	1.26
	Apidae	*Apis mellifera*	27	25	28	80	50.31
	Colletidae	*Colletes* spp.	0	0	1	1	0.63
	Halictidae	*Halictus* spp.	1	0	1	2	1.26
		*Lasioglossum* spp.	2	9	10	21	13.21
		*Nomioides* spp.	0	1	0	1	0.63
	Megachilidae	*Anthidium* spp.	0	2	2	4	2.52
		*Hoplitis* spp.	0	1	0	1	0.63
		*Megachile* spp.	2	5	2	9	5.66
		Megach. Un.	1	0	0	1	0.63
		*Rhodanthidium* spp.	1	0	0	1	0.63
		*Stelis* spp.	0	0	1	1	0.63
	wild Apidae	*Ceratina* spp.	3	1	5	9	5.66
		*Nomada* spp.	1	0	0	1	0.63
		Wildbee Un.	1	0	2	3	1.89
Other Hymenoptera	Hymenoptera		1	0	3	4	2.52
	Vespoidea		1	1	0	2	1.26
Lepidoptera	Lepidoptera		0	1	3	4	2.52
		*Pieris* spp.	1	1	3	5	3.14
	Satyrinae		0	0	1	1	0.63

**Table 3:** List of genera observed in pan traps on the melon crops with managed herbaceous (MH), unmanaged herbaceous (UH), and managed shrubby (MS) margins. %: percentage of captures concerning the total of pollinator insects registered. Un. unidentified.

Order	Suborder/Family	Genera	Treatment			Total	%
			MS	UH	MH		
Araneae			6	1	1	8	0.30
Diptera	Dipt. Un.		8	13	17	38	1.42
	Syrphidae		125	46	63	234	8.73
Heteroptera			11	0	0	11	0.41
Hymenoptera	Chrysididae		2	0	0	2	0.07
	Crabronidae	*Oxybellus* sp.	0	0	1	1	0.04
	Symphita		1	1	0	2	0.07
	Hym. Un.		263	122	166	551	20.55
Hymenoptera (Apoidea)	Andrenidae	*Andrena* sp.	43	24	32	99	3.69
		*Panurgus* sp.	5	4	7	16	0.60
	Apidae	*Apis mellifera*	126	136	87	349	13.02
		*Bombus* sp.	6	3	3	12	0.45
		*Amegilla* sp.	0	1	0	1	0.04
		*Ceratina* sp.	1	1	1	3	0.11
		*Eucera* sp.	93	23	22	138	5.15
		*Nomada* sp.	1	1	1	3	0.11
		*Xylocopa* sp.	1	0	1	2	0.07
	Colletidae	*Colletes* sp.	6	1	1	8	0.30
		*Hylaeus* sp.	20	19	15	54	2.01
	Halictidae	*Ceylalictus* sp.	2	0	0	2	0.07
		*Dufourea* sp.	0	0	1	1	0.04
		*Halictus* sp.	36	24	24	84	3.13
		*Lasioglossum* sp.	270	296	374	940	35.06
		*Nomia* sp.	1	1	1	3	0.11
		*Nomioides* sp.	37	27	18	82	3.06
	Megachilidae	*Anthidium* sp.	2	1	1	4	0.15
		*Heriades* sp.	2	1	3	6	0.22
		*Megachile* sp.	3	2	3	8	0.30
		*Osmia* sp.	3	1	3	7	0.26
		*Pseudoanthidium* sp.	1	0	1	2	0.07
		*Stelis* sp.	0	2	0	2	0.07
Lepidoptera			4	0	0	4	0.15

## Data Availability

Data are contained within the article.

## Supplementary Materials

The following supporting information can be downloaded at: https://www.mdpi.com/article/10.3390/insects14030296/s1, Table S1: Species of plants sampled in the three treatments. The species sown or transplanted in managed herbaceous and shrubby margins are shown in bold; the rest of the species were naturally emerged plants. UH: unmanaged herbaceous margins; MH: managed herbaceous margins; MS: managed shrubby margins. Table S2: Results of GLMM for the effect of the type of floral margin, year, their interaction (type of floral margin × year), and distance from the margin on the abundance of the different bee groups in pan traps on melon crops. df: degrees of freedom.
