# Supplementary material for: Effects of Managed and Unmanaged Floral Margins on Pollination Services and Production in Melon Crops
Source: Insects. 2023 Mar 20;14(3):296. doi: 10.3390/insects14030296 (PMC10051670; doi:10.3390/insects14030296)
Supplement: Supplementary file 1 [file insects-14-00296-s001.zip › insects-2244622-supplementary.pdf]

Supplementary Material

Table S1. Species of plants sampled in the three treatments. The species sown or transplanted in managed herbaceous and shrubby margins are shown in bold; the rest of the species were naturally emerged plants. UH: unmanaged herbaceous margins; MH: managed herbaceous margins; MS: managed shrubby margins.

Family	Plant Species	Margin		
		UH	MH	MS
Apiaceae	*Conium* sp.	x	x	
	Coriandrum sativum	x	x	
	Foeniculum vulgare	x		
Asteraceae	*Anacyclus* sp.	x	x	
	Asteraceae	x	x	
	Calendula arvensis	x	x	
	Calendula sp.	x	x	
	Carduus sp.	x	x	
	Chrysanthemum coronarium	x	x	
	Matricaria chamomilla		x	
	Santolina chamaecyparisus			x
	Sonchus spp.	x	x	
Boraginaceae	***Borago officinalis***		x	
	Echium vulgare	x	x	
Brassicaceae	*Brassica* sp.	x	x	
	Brassicaceae	x		
	Carrichtera annua	x	x	
	Diplotaxis catholica		x	
	Diplotaxis erucoides	x	x	
	Eruca vesicaria	x	x	
	Moricandia arvensis	x	x	
	Rapistrum sp.	x	x	
	Sisymbrium sp.	x	x	
Caryophyllaceae	***Silene vulgaris***		x	
Convolvulaceae	*Convolvulus* sp.	x	x	
Fabaceae	***Dorycnium pentaphyllum***			x
	Medicago sp.		x	
	Melilotus officinalis	x	x	
	Vicia sativa		x	
Lamiaceae	***Ballota hirsuta***			x
	Lamium sp.		x	
	Lavandula dentata			x
	Lavandula stoechas			x
	***Marrubium* sp**	x		
	Phlomis purpurea			x
	Rosmarinus officinalis			x
	Salvia officinalis			x
	Salvia verbenaca		x	
	Thymus mastichina			x
Ranunculaceae	***Nigella damascena***		x	

Table S2. Results of GLMM for the effect of the type of floral margin, year, their interaction (type of floral margin*year), and distance from the margin on the abundance of the different bee groups in pan traps on melon crops. df: degrees of freedom.

	Treatment			Year			Treatment*Year			Distance		
Group	χ^{2-}-value	df	P-value	χ^2-value	df	P-value	χ^2-value	df	P-value	χ^2-value	df	P-value
Andrenidae	1.0	2	0.602	0.3	1	0.590	30.8	2	<0.001	7.3	1	0.007
A. mellifera	6.6	2	0.036	1.0	1	0.308	5.5	2	0.063	0.2	1	0.667
Apidae	32.8	2	<0.001	0.6	1	0.453	2.8	2	0.249	4.9	1	0.026
Colletidae	1.5	2	0.458	8.4	1	0.003	4.3	2	0.115	0.5	1	0.473
Halictidae	2.3	2	0.319	26.3	1	<0.001	5.0	2	0.081	0.4	1	0.543
Megachilidae	0.7	2	0.688	1.2	1	0.281	8.6	2	0.013	0.3	1	0.590
